# Supplementary figures and images for: The relationship between family conflict resolution methods and depressive symptoms in patients with chronic diseases
Source: PLoS One. 2025 Feb 28;20(2):e0318378. doi: 10.1371/journal.pone.0318378 (PMC11870374; doi:10.1371/journal.pone.0318378)

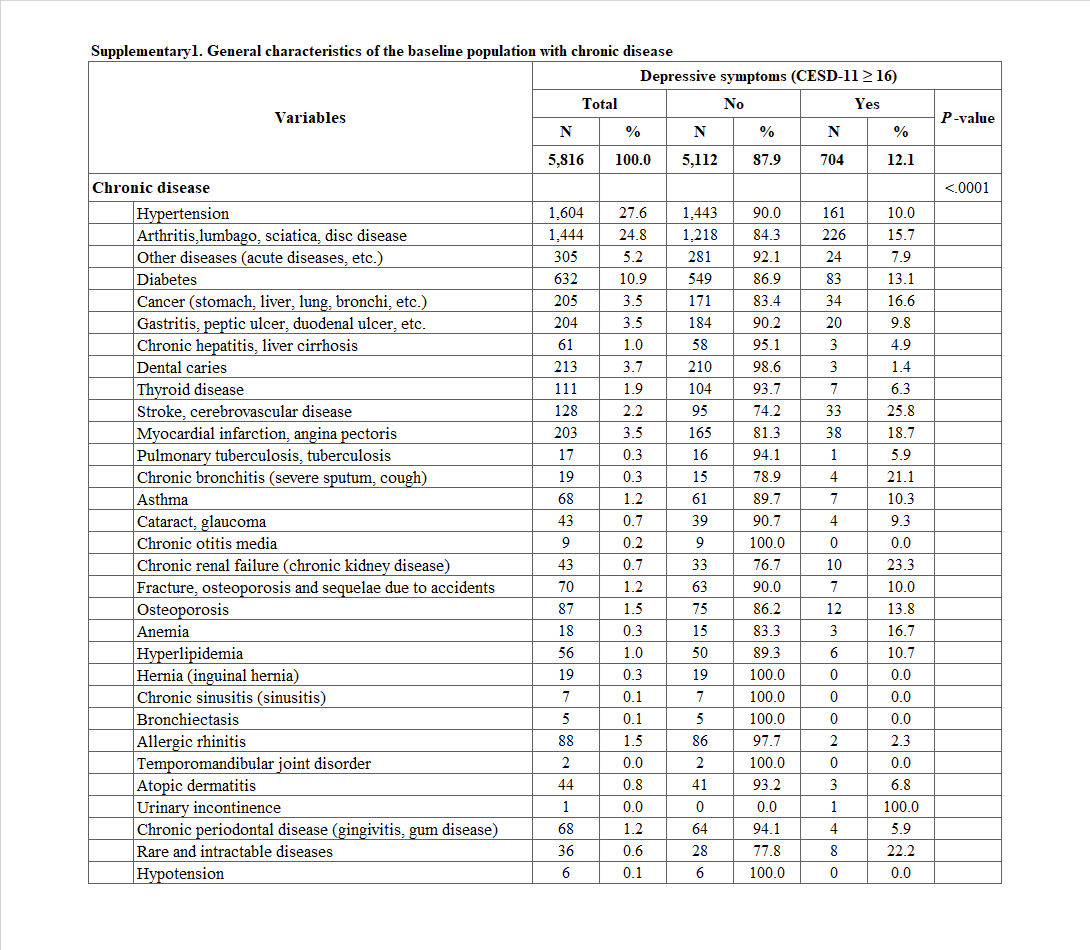

Supplement: S1 Table — (TIF) [file pone.0318378.s001.tif]
