# Supplementary material for: The relationship between family conflict resolution methods and depressive symptoms in patients with chronic diseases
Source: PLoS One. 2025 Feb 28;20(2):e0318378. doi: 10.1371/journal.pone.0318378 (PMC11870374; doi:10.1371/journal.pone.0318378)
Supplement: Attachment — Submitted filename: Response to Reviewers_PONE-D-24-06786.pdf [file pone.0318378.s003.pdf]

Revision Note for ID PONE-D-24-06786

"The relationship between family conflict resolution methods and depressive symptoms in patients with chronic diseases”

We were pleased to have the opportunity to revise our paper. In revising our paper, we have carefully considered your comments and suggestions. As instructed, we have attempted to explain the changes made in reaction to all the reviewers’ comments. The reviewers’ comments were very helpful overall, and we appreciate the constructive feedback on our original submission. After addressing the issues raised, we feel the quality of the paper has greatly improved and we hope you agree. Our response to each comment is as follows, and we attach a revision note with the highlighted, revised sections of the manuscript. Again, thank you for the valuable and helpful comments.

Response to Editorial Board Member’s comments

Thank you for submitting your manuscript to PLOS ONE. After careful consideration, we feel that it has merit but does not fully meet PLOS ONE’s publication criteria as it currently stands. Therefore, we invite you to submit a revised version of the manuscript that addresses the points raised during the review process.

- ➔ *Thank you for your great efforts in reviewing our manuscript. The authors will cautiously read your comments and take meaningful suggestions into our consideration. After the review, we totally revised our manuscript. Our response to each comment is as follows;*

Response to Editor Comments:

The study examined an important area of research, but the authors should address the suggested comments before considering publication. Address all comments raised by both reviewers

➔ *Thank you for your great efforts in reviewing our manuscript. The authors will cautiously read your comments and take meaningful suggestions into our consideration. After the review, we totally revised our manuscript.*

Point 1

In the methods section, use a diagram that is clear and easy for readers to understand how the sample used for the current analyses was selected from the total eligible sample.

Response 1: *Thank you for your meaningful comment. we created a flow chart on how the participant sample was selected. We excluded participants who participated from 2012 to 2022 if they did not have chronic diseases, were not married or did not have cohabitants, were diagnosed with depression, or showed depressive symptoms in their first entry year in the panel. At baseline, there were a total of 10,969 participants. The average participation period for the panel participants was 5.4 years.*

- ✓ ***Revised manuscript, line93-99, page 4, Methods:*** ***For our study's data, the presence of chronic diseases was reported by participants themselves. We categorized participants as chronic disease patients if they reported having the condition for less than 3 months, between 3 months and less than 6 months, or more than 6 months [23]. We excluded participants from 2012 to 2022 who were younger than 20 years old, did not have chronic diseases, were not married or did not have cohabitants, were diagnosed with depression, or showed depressive symptoms in their first entry year in the panel. At baseline, there were a total of 5,816 participants. The average participation period for the panel participants was 5.4 years.***

- ✓ ***Added references****:*

- *Han K-M, Ko Y-H, Yoon H-K, Han C, Ham B-J, Kim Y-K. Relationship of depression, chronic disease, self-rated health, and gender with health care utilization among community-living elderly. Journal of Affective Disorders. 2018;241:402-10.*

✓ ***Revised manuscript, Results:*** *Update Figure 1. Participation flow chart*

Fig 1. Flow chart of participant selection.

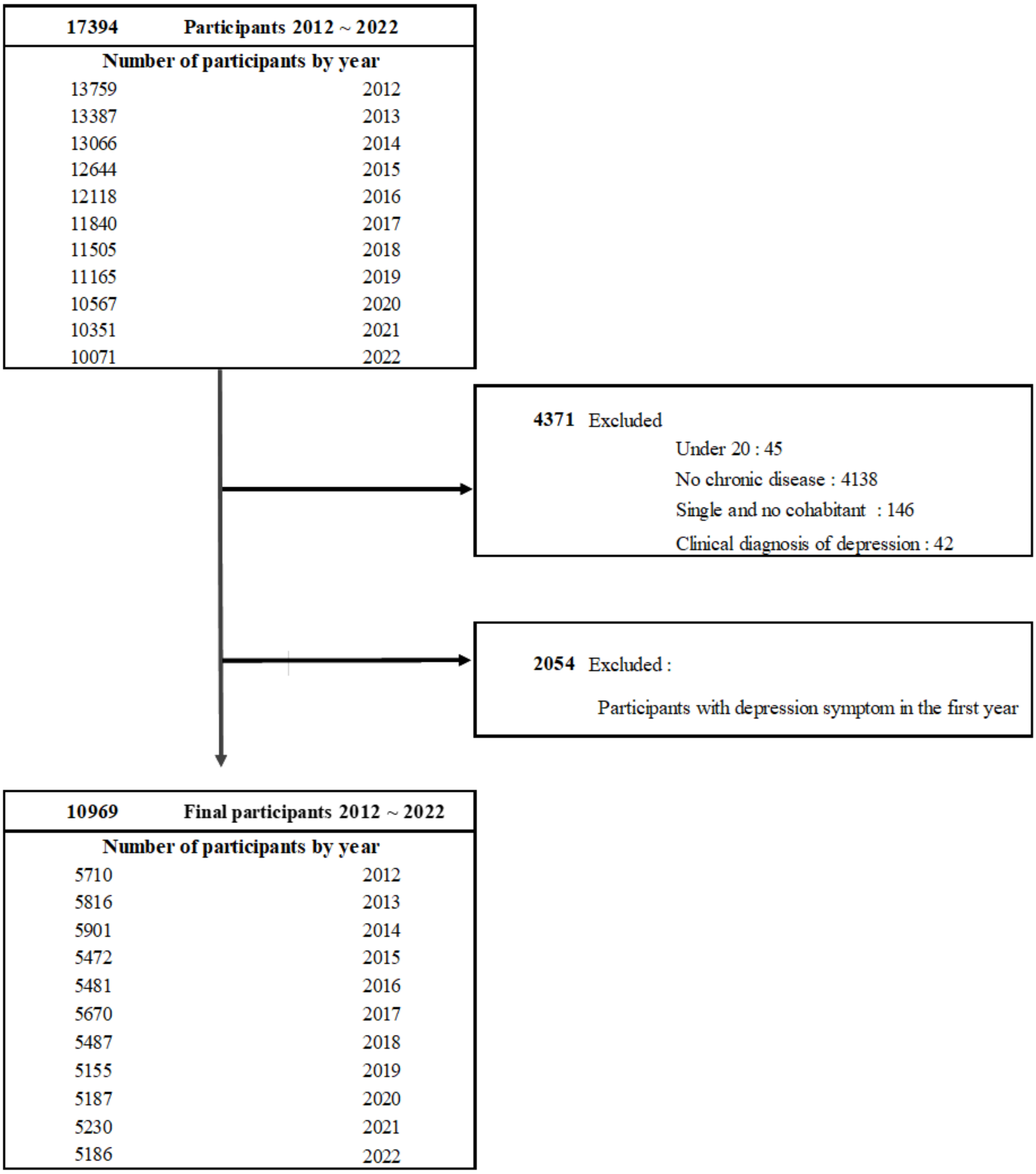

Point 2

Explain more detail how the family conflict resolution method was analysed (e.g. how consecutive years were considered to decide whether family conflict resolution is positive/negative?)

Response 2: *Thank you for your comment. We appreciate your comment very much. The approach to handling family conflict is based on the scoring and evaluation method suggested by KOWEP. In this study, we evaluated how family members have interacted and resolved conflicts over the past year based on participants' subjective responses to five items. Participants responded to the items using a scale of "Not at all," "Somewhat disagree," "Neutral," "Somewhat agree," and "Strongly agree." Scores are assigned to each item based on the responses, and higher total scores indicate a more positive assessment. For the statements "There are frequent disagreements in my family," "My family members get so angry that they throw things," "My family members criticize each other," and "My family members hit each other," a response of "Not at all" is given 5 points, and a response of "Strongly agree" is given 1 point. For the statement "My family members discuss issues calmly," a response of "Not at all" is given 1 point, and a response of "Strongly agree" is given 5 points. The calculated values are then divided by 5 for evaluation. In order to establish categorical thresholds for resolving family conflicts, the responses were grouped into two distinct groups based on the median of the number of responses. Similarly, categorical thresholds were determined for each question based on the median value. Responses exceeding the median were classified as positive approaches to conflict resolution, while those falling below the median were classified as negative approaches. We were able to improve our manuscript based on the meaningful comments that you have given us. Once again, thank you for giving us meaningful feedback.*

- ✓ ***Revised manuscript, line 111-126, page 6-7, Methods Data source and study population :** In this study, the main independent variable was the methods for managing family conflict, evaluated using the scoring and evaluation method recommended by KoWePS. We evaluated how family members have interacted and resolved conflicts over the past year based on participants' subjective responses to five items. Participants responded to the items using a scale of "Not at all,"*

"Somewhat disagree," "Neutral," "Somewhat agree," and "Strongly agree." Scores are assigned to each item based on the responses, and higher total scores indicate a more positive assessment. For the statements "There are frequent disagreements in my family," "My family members get so angry that they throw things," "My family members criticize each other," and "My family members hit each other," a response of "Not at all" is given 5 points, and a response of "Strongly agree" is given 1 point. For the statement "My family members discuss issues calmly," a response of "Not at all" is given 1 point, and a response of "Strongly agree" is given 5 points. The calculated values are then divided by 5 for evaluation. In order to establish categorical thresholds for resolving family conflicts, the responses were grouped into two distinct groups based on the median of the number of responses. Similarly, categorical thresholds were determined for each question based on the median value. Responses exceeding the median were classified as positive approaches to conflict resolution, while those falling below the median were classified as negative approaches.

Point 3

Clearly label outcome, exposure and confounding variables in the methods section.

Response 3: *Thank you for your positive feedback on our study. We fully agree with you. We fully agree with you. We will provide clearer labeling for the outcome, exposure, and confounding variables in the methods section. Furthermore, we will provide more detailed explanations regarding the criteria used to divide the variables into categories.*

- ✓ ***Revised manuscript, line 102-139, page 6-7, Methods Measure:*** ***The dependent variable was depressive symptoms. The research employed the Center for Epidemiologic Studies Depression Scale (CESD-11) to assess depressive symptoms [21]. The CESD-11, a concise version of the original 20-item scale, is a well-validated self-report screening instrument. The total CESD-11 score is computed by summing the scores for all 11 questions and multiplying them by 20/11 [22, 23]. A score of 16 or higher indicates the presence of depressive symptoms; scores falling within the range of 16–20 indicate mild depressive symptoms; scores between 20 and***

24 indicate moderate depressive symptoms and a score of 25 or higher indicates severe depressive symptoms.

In this study, the main independent variable is the methods for managing family conflict, evaluated using the scoring and evaluation method recommended by KOWEP. We evaluated how family members have interacted and resolved conflicts over the past year based on participants' subjective responses to five items. Participants responded to the items using a scale of "Not at all," "Somewhat disagree," "Neutral," "Somewhat agree," and "Strongly agree." Scores are assigned to each item based on the responses, and higher total scores indicate a more positive assessment. For the statements "There are frequent disagreements in my family," "My family members get so angry that they throw things," "My family members criticize each other," and "My family members hit each other," a response of "Not at all" is given 5 points, and a response of "Strongly agree" is given 1 point. For the statement "My family members discuss issues calmly," a response of "Not at all" is given 1 point, and a response of "Strongly agree" is given 5 points. The calculated values are then divided by 5 for evaluation. In order to establish categorical thresholds for resolving family conflicts, the responses were grouped into two distinct groups based on the median of the number of responses. Similarly, categorical thresholds were determined for each question based on the median value. Responses exceeding the median were classified as positive approaches to conflict resolution, while those falling below the median were classified as negative approaches

We controlled for potential confounding variables in our study [24]. Socio-demographic factors included sex, age (19–29 / 30–39 / 40–49 / 50–59 / ≥ 60), region (metropolitan/non-metropolitan areas), household income (based on adjusted gross income below 60% of median as low/60% and above as normal), economic activities (yes/no), and education level (college or higher/high school/middle school or lower). Health-related factors included smoking (yes/no), current alcohol use (yes/no), and self-perceived health status (good/normal/bad). We also included type of chronic disease and family relationship satisfaction as variables. Chronic disease data from

KoWePS were used to classify the 32 selected chronic diseases into the top 4 most frequently reported primary conditions(excluding depression) and others (hypertension/arthritis/acute conditions/ diabetes/other chronic diseases). The variable for family relationship satisfaction was categorized based on personal satisfaction with family relationships over the past year (dissatisfaction/satisfaction). All responses were self-reported and collected according to the format specified by KoWePS.

✓ ***Added references***:

- Chon k-K, Rhee M-K. Preliminary Development of Korean Version of CES-D. Korean Journal of Clinical Psychology. 1992;11(1):65-76.

- Kang SH, Kim S, Park E-C, Jang S-I. Effects of material hardship on depression among adults in South Korea: insights from by the Korea Welfare Panel Study 2008–2017. International Journal for Equity in Health. 2021;20(1):202. doi: 10.1186/s12939-021-01531-1.

- Shin J, Choi JW, Jang S-i, Choi Y, Lee SG, Ihm TH, et al. The temporal association of excessive health expenditure with suicidal ideation among primary income earners: a cross-sectional design using the Korean Welfare Panel Survey (KoWePS). BMJ Open. 2015;5(6):e007421. doi: 10.1136/bmjopen-2014-007421.

- Kim JY. Does poverty matter for depression in people with disabilities? The Mediating Effects of Life Satisfaction. Journal of Social Service Research. 2022;48(3):430-9.

- Ma S, Jeon B, Roh S-h, Kim C-S. Violence victimization experiences and depression among individuals with disabilities. Korean J Health Educ Promot. 2023;40(5):55-68.

Point 4

Also, provide more information on the instruments used to assess some of the variables, such as income (low/normal?), family life, health status, smoking (current/ever?), drinking (alcohol or what?), etc.

Response 4: *Thank you for your positive feedback on our study. We agree with the suggestion for additional explanations about the variables, and have incorporated further information regarding each variable. Thanks again your great effort in reviewing our manuscript.*

- ✓ ***Revised manuscript, line 127-139, page 7, Methods Measure:***

  We controlled for potential confounding variables in our study [24]. Socio-demographic factors included sex, age (19–29 / 30–39 / 40–49 / 50–59 / ≥ 60), region (metropolitan/non-metropolitan areas), household income (based on adjusted gross income below 60% of median as low/60% and above as normal), economic activities (yes/no), and education level (college or higher/high school/middle school or lower). Health-related factors included smoking (yes/no), current alcohol use (yes/no), and self-perceived health status (good/normal/bad). We also included type of chronic disease and family relationship satisfaction as variables. Chronic disease data from KoWePS were used to classify the 32 selected chronic diseases into the top 4 most frequently reported primary conditions(excluding depression) and others (hypertension/arthritis/acute conditions/ diabetes/other chronic diseases). The variable for family relationship satisfaction was categorized based on personal satisfaction with family relationships over the past year (dissatisfaction/satisfaction). All responses were self-reported and collected according to the format specified by KoWePS.

Point 5

Make major revisions to the data presented in all tables (N(%), 95%CI, age category?, p-value for age in table 1, include % for all frequencies across all rows, p-value for chronic disease?, be consistent with decimals across all tables (e.g. <.0001, 0.0007), keep journal rule for spacing, table 2 95% CI should be revised).

Response 5: *Thank you for your meaningful comment. We fully agree with you that we need to adhere to the table regulations. We have modified the table format accordingly and standardized the decimal points. Additionally, we have included the previously missing information. We would be grateful if you recheck the manuscript and if there is anything lacking or that needs to be corrected, I will revise again it right away. Once again, thank you for your great effort in reviewing our manuscript and giving meaningful feedback.*

- ✓ ***Revised manuscript, Results:* Update Table 1, Table 2, Table 3, Figure 2.**

✓ *Table1. General characteristics of the baseline population.*

Table 1. General characteristics of the baseline population

Variables		Depressive symptoms (CESD-11 ≥ 16)						
		Total		No		Yes		*P* -value
		N	%	N	%	N	%	
		**5,816**	**100.0**	**5,112**	**87.9**	**704**	**12.1**	
**Family conflict resolution methods**								<.0001
	Positive →Positive	1,866	32.1	1710	91.6	156	8.4	
	Negative→Positive	1,214	20.9	1077	88.7	137	11.3	
	Positive →Negative	1,049	18.0	913	87.0	136	13.0	
	Negative→Negative	1,687	29.0	1412	83.7	275	16.3	
**Sex**								<.0001
	Male	2,442	42.0	2242	91.8	200	8.2	
	Female	3,374	58.0	2870	85.1	504	14.9	
**Age**								<.0001
	20-29	133	2.3	130	97.7	3	2.3	
	30-39	284	4.9	275	96.8	9	3.2	
	40-49	557	9.6	532	95.5	25	4.5	
	50-59	958	16.5	881	92.0	77	8.0	
	≤60	3,884	66.8	3294	84.8	590	15.2	
**Region**								0.2548
	Metropolitan	4,348	74.8	3834	88.2	514	11.8	
	Non-metropolitan	1,468	25.2	1278	87.1	190	12.9	
**Household income**								<.0001
	Normal	3,370	57.9	3151	93.5	219	6.5	
	Low	2,446	42.1	1961	80.2	485	19.8	
**Economic activity**								<.0001
	No	2,498	43.0	2277	91.2	221	8.8	
	Yes	3,318	57.0	2835	85.4	483	14.6	
**Educational level**								<.0001
	Middle school or lower	3,606	62.0	3030	84.0	576	16.0	
	High school	1,341	23.1	1252	93.4	89	6.6	
	College or higher	869	14.9	830	95.5	39	4.5	
**Smoking**								0.0916
	No	4,949	85.1	4335	87.6	614	12.4	
	Yes	867	14.9	777	89.6	90	10.4	
**Current Alcohol Use**								<.0001
	No	3,494	60.1	2953	84.5	541	15.5	
	Yes	2,322	39.9	2159	93.0	163	7.0	
**Health status**								<.0001
	Bad	1,895	32.6	1421	75.0	474	25.0	
	Normal	1,818	31.3	1674	92.1	144	7.9	
	Good	2,103	36.2	2017	95.9	86	4.1	
**Family relationship**								<.0001
	Dissatisfaction	1,157	19.9	869	75.1	288	24.9	
	Satisfaction	4,659	80.1	4243	91.1	416	8.9	
**Chronic disease**								<.0001
	Hypertension	1,604	27.6	1,443	90.0	161	10.0	
	Arthritis	1,444	24.8	1,218	84.3	226	15.7	
	Acute diseases	305	5.2	281	92.1	24	7.9	
	Diabetes	632	10.9	549	86.9	83	13.1	
	Other Chronic diseases	1,831	31.5	1621	88.5	210	11.5	

✓ ***Table 2. Unadjusted and adjusted modal for depressive symptoms with change of family conflict resolution methods in 2012-2022.***

Table 2. Unadjusted and adjusted modal for depressive symptoms with change of family conflict resolution methods in 2012 to 2022.

Variables[a]		Depressive symptoms (CESD-11 ≥ 16)			
		OR	95%CI		
**Family conflict resolution methods**					
**Model I[a]**					
	Positive →Positive	1.00			
	Negative→Positive	1.23	(1.15	-	1.32)
	Positive →Negative	1.55	(1.45	-	1.66)
	Negative→Negative	1.88	(1.75	-	2.02)
**Model II[b]**					
	Positive →Positive	1.00			
	Negative→Positive	1.22	(1.13	-	1.31)
	Positive →Negative	1.53	(1.43	-	1.64)
	Negative→Negative	1.82	(1.69	-	1.96)
**Model III[c]**					
	Positive →Positive	1.00			
	Negative→Positive	1.14	(1.05	-	1.22)
	Positive →Negative	1.34	(1.24	-	1.44)
	Negative→Negative	1.48	(1.37	-	1.59)

[a]Adjusted for Sex, Age ;

[b]Additinally adjusted for region, household income, economic activities, education level on the base of Model 1.

[c]Additinally adjusted for smoking, current alcohol use, health status, type of chronic disease, family relationship on the base of Model 2.

Table 3. Subgroup analysis using the generalized estimating equation depressive symptom among chronic patients with family conflict resolution in 2012-2022

Table 3. Subgroup analysis using the generalized estimating equation depressive symptom among chronic patients with family conflict resolution in 2012-2022

**Variables[a]**		**Depressive symptoms (CESD-11 ≥ 16)**													
		**Family conflict resolution methods**													
		**Positive →Positive**		**Negative→Positive**				**Positive →Negative**				**Negative→Negative**			
		**OR**		**OR**	**95% CI**			**OR**	**95% CI**			**OR**	**95% CI**		
**Sex**															
	Male	1.00		1.24	(1.09	-	1.42)	1.44	(1.25	-	1.65)	1.57	(1.37	-	1.80)
	Female	1.00		1.09	(1.00	-	1.19)	1.29	(1.18	-	1.42)	1.44	(1.32	-	1.58)
**Age**															
	20-29	1.00		-				-				-			
	30-39	1.00		0.80	(0.45	-	1.44)	1.11	(0.58	-	2.14)	1.17	(0.66	-	2.08)
	40-49	1.00		1.36	(0.89	-	2.10)	1.27	(0.83	-	1.96)	1.97	(1.31	-	2.95)
	50-59	1.00		1.23	(0.95	-	1.60)	1.46	(1.14	-	1.88)	1.96	(1.55	-	2.47)
	60≤	1.00		1.12	(1.04	-	1.22)	1.34	(1.23	-	1.45)	1.41	(1.30	-	1.53)
**Region**															
	Metropolitan	1.00		1.17	(1.07	-	1.28)	1.37	(1.25	-	1.49)	1.52	(1.39	-	1.66)
	Non-metropolitan	1.00		1.07	(0.93	-	1.22)	1.28	(1.11	-	1.48)	1.38	(1.19	-	1.59)
**Household income**															
	Normal	1.00		1.13	(1.00	-	1.28)	1.31	(1.15	-	1.48)	1.65	(1.46	-	1.86)
	Low	1.00		1.13	(1.03	-	1.24)	1.36	(1.24	-	1.49)	1.38	(1.25	-	1.51)
**Economic activity**															
	No	1.00		1.17	(1.07	-	1.28)	1.29	(1.17	-	1.41)	1.40	(1.28	-	1.53)
	Yes	1.00		1.06	(0.93	-	1.21)	1.42	(1.25	-	1.62)	1.62	(1.43	-	1.84)
**Educational level**															
	Middle school or lower	1.00		1.10	(1.00	-	-	1.50	(1.36	-	-	1.55	(1.40	-	-
	High school	1.00		1.22	(1.01	-	1.46)	1.21	(1.00	-	1.46)	1.53	(1.28	-	1.84)
	College or higher	1.00		1.19	(0.92	-	1.54)	1.33	(1.03	-	1.72)	1.84	(1.43	-	2.37)
**Smoking**															
	No	1.00		1.13	(1.04	-	1.22)	1.37	(1.27	-	1.48)	1.48	(1.36	-	1.60)
	Yes	1.00		1.15	(0.91	-	1.47)	1.06	(0.82	-	1.36)	1.41	(1.11	-	1.79)
**Current Alcohol Use**															
	No	1.00		1.12	(1.03	-	1.21)	1.31	(1.21	-	1.43)	1.41	(1.29	-	1.53)
	Yes	1.00		1.21	(1.03	-	1.42)	1.47	(1.25	-	1.73)	1.68	(1.44	-	1.96)
**Health status**															
	Bad	1.00		1.08	(0.98	-	1.19)	1.30	(1.17	-	1.43)	1.28	(1.16	-	1.41)
	Normal	1.00		1.21	(1.06	-	1.40)	1.31	(1.13	-	1.51)	1.49	(1.31	-	1.70)
	Good	1.00		1.20	(0.98	-	1.46)	1.51	(1.24	-	1.83)	2.07	(1.74	-	2.46)
**Family relationship**															
	Dissatisfaction	1.00		1.10	(0.93	-	1.29)	1.25	(1.08	-	1.45)	1.49	(1.30	-	1.72)
	Satisfaction	1.00		1.14	(1.05	-	1.24)	1.37	(1.25	-	1.49)	1.39	(1.27	-	1.52)
**Chronic disease**															
	Hypertension	1.00		-				-				-			
	Arthritis	1.00		1.13	(0.95	-	1.33)	1.37	(1.15	-	1.64)	1.46	(1.24	-	1.73)
	Acute diseases	1.00		0.86	(0.53	-	1.41)	1.38	(0.88	-	2.17)	1.71	(1.13	-	2.57)
	Diabetes	1.00		1.04	(0.81	-	1.35)	1.47	(1.15	-	1.89)	1.45	(1.15	-	1.82)
	Other Chronic diseases	1.00		1.13	(1.02	-	1.25)	1.23	(1.11	-	1.36)	1.45	(1.31	-	1.61)

[a]Adjusted for all covariates

Figure 2. Results of sensitivity analysis stratified by interesting variables in 2012 to 2022.

Fig 2. Results of sensitivity analysis stratified by interesting variables in 2012 to 2022.

Variables[a]		Depressive symptoms (CESD-11 ≥ 16) OR (95%CI)
**Frequent conflicts of opinion**		
	Positive →Positive	
	Negative→Positive	
	Positive →Negative	
	Negative→Negative	
**Throwing object**		
	Positive →Positive	
	Negative→Positive	
	Positive →Negative	
	Negative→Negative	
**Collaborative discussion**		
	Positive →Positive	
	Negative→Positive	
	Positive →Negative	
	Negative→Negative	
**Imputation of each other**		
	Positive →Positive	
	Negative→Positive	
	Positive →Negative	
	Negative→Negative	
**Domestic violence**		
	Positive →Positive	
	Negative→Positive	
	Positive →Negative	
	Negative→Negative	

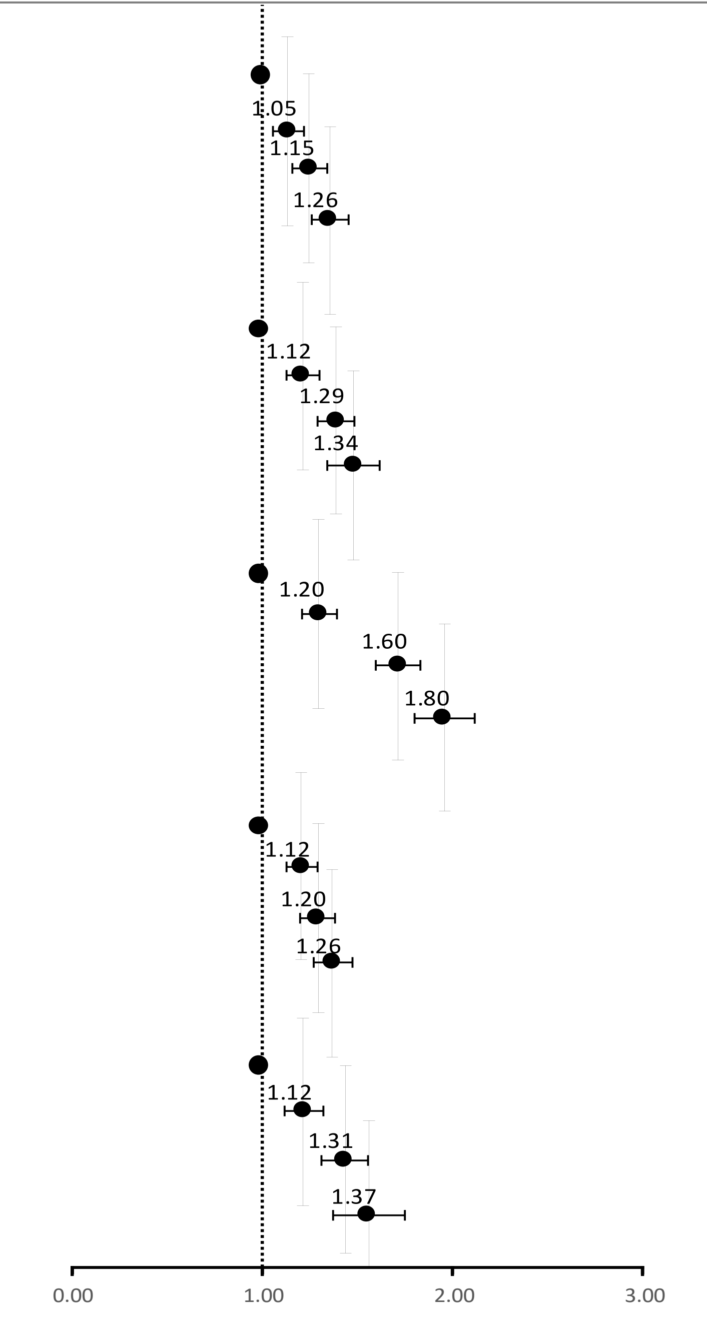

[a]Adjusted for all covariates

Point 6

In Table 2, what is the point of reporting the OR of other confounders when your exposure variable (EV) is family conflict resolution method? Instead, present the OR of the EV in different models (Model I (unadjusted), Model II, Model III (fully adjusted)) and report the adjusted variables as a footnote below the table.

Response 6: *Thank you for your meaningful comment. We have divided Table 2 into models 1, 2, and 3 for analysis as per your suggestion. Model 1 is an unadjusted model that includes only sex and age. Model 2 includes additional analysis with socio-demographic variables such as region, household income, economic activities, and education level. Model 3 is a fully adjusted model that accounts for health factors such as smoking, current alcohol use, health status, type of chronic disease, and family relationship. We would be grateful if you recheck the manuscript and if there is anything lacking or that needs to be corrected, I will revise again it right away. Once again, thank you for your great effort in reviewing our manuscript and giving meaningful feedback.*

- ✓ ***Revised manuscript, Results:** Update Table 2*

✓ ***Table 2. Unadjusted and adjusted modal for depressive symptoms with change of family conflict resolution methods in 2012-2022.***

Table 2. Unadjusted and adjusted modal for depressive symptoms with change of family conflict resolution methods in 2012 to 2022.

Variables[a]		Depressive symptoms (CESD-11 ≥ 16)			
		OR	95%CI		
**Family conflict resolution methods**					
**Model I[a]**					
	Positive →Positive	1.00			
	Negative→Positive	1.23	(1.15	-	1.32)
	Positive →Negative	1.55	(1.45	-	1.66)
	Negative→Negative	1.88	(1.75	-	2.02)
**Model II[b]**					
	Positive →Positive	1.00			
	Negative→Positive	1.22	(1.13	-	1.31)
	Positive →Negative	1.53	(1.43	-	1.64)
	Negative→Negative	1.82	(1.69	-	1.96)
**Model III[c]**					
	Positive →Positive	1.00			
	Negative→Positive	1.14	(1.05	-	1.22)
	Positive →Negative	1.34	(1.24	-	1.44)
	Negative→Negative	1.48	(1.37	-	1.59)

[a]Adjusted for Sex, Age ;

[b]Additinally adjusted for region, household income, economic activities, education level on the base of Model 1.

[c]Additinally adjusted for smoking, current alcohol use, health status, type of chronic disease, family relationship on the base of Model 2.

Point 7

Delete a figure presented before the discussion. I think the result presented in Table 5 does not add anything to the study as the association between category of family conflict resolution method and depression is significant at almost all levels. Significantly revise the discussion, add more scientific theories to support your argument, use updated literature and include the policy and research implications of the study findings. Revise and update the conclusion accordingly.

Response 7: *Thank you for your meaningful comment. We fully agree with your suggestion. First, we will delete Table 5 as it is deemed unnecessary. Additionally, we have removed unnecessary parts from the discussion section and added the aspects we wanted to emphasize from our findings. Your valuable feedback has helped us improve our manuscript. Thank you.*

- ✓ ***Revised manuscript, line 199-215, page 16, Discussion:* Our study found an association between depressive symptoms and changes in how patients with chronic diseases resolve family conflicts. A change in the negative approach to resolving conflict and no change in the negative approach were linked to higher odds of experiencing depressive symptoms compared with the positive approach. By contrast, participants who changed from a negative to a positive method showed lower levels of depressive symptoms than those who continued using a negative method of conflict resolution. Consistent with previous studies, our results indicated that conflict within family relationships influenced depressive symptoms, and addressing conflicts is crucial for managing such symptoms [30, 31].**

  Our study revealed that economically active participants with chronic diseases faced higher odds of depressive symptoms when their family conflict resolution methods remained persistently negative. This finding aligns with existing research indicating that individuals with chronic diseases are more susceptible to presenteeism [32] and psychological stress in the workplace compared to healthy individuals [33], which can spill over into the home, exacerbating family relationship conflicts [34]. Therefore, addressing mental health issues for individuals with chronic diseases is crucial in both workplace and home settings [35]. Our study findings are thus corroborated by those of other studies. This underscores the need for continuous

attention and support to improve the overall mental health of individuals with chronic diseases.

✓ ***Added references:***

- Holland P, Collins AM. "Whenever I can I push myself to go to work": a qualitative study of experiences of sickness presenteeism among workers with rheumatoid arthritis. Disability and Rehabilitation. 2018;40(4):404-13. doi: 10.1080/09638288.2016.1258436.

- McGonagle AK, Barnes-Farrell JL. Chronic Illness in the Workplace: Stigma, Identity Threat and Strain. Stress and Health. 2014;30(4):310-21. doi: https://doi.org/10.1002/smi.2518.

- Frone MR, Russell M, Cooper ML. Relation of work–family conflict to health outcomes: A four-year longitudinal study of employed parents. Journal of Occupational and Organizational Psychology. 1997;70(4):325-35. doi: https://doi.org/10.1111/j.2044-8325.1997.tb00652.x.

- Sorensen G, Landsbergis P, Hammer L, III BCA, Linnan L, Yancey A, et al. Preventing Chronic Disease in the Workplace: A Workshop Report and Recommendations. American Journal of Public Health. 2011;101(S1):S196-S207. doi: 10.2105/ajph.2010.300075. PubMed PMID: 21778485.

Response to Reviewer #1's comments

This was a really interesting topic which addressed how family conflict resolve methods may affect depression among chronic disease patients who are likely to be more depressive than those disease-free individuals. They did careful analyses, however I have following questions, hopefully they can be addressed by investigators.

➔ *Thank you for your great efforts in reviewing our manuscript. The authors will cautiously read your comments and take meaningful suggestions into our consideration. After the review, we totally revised our manuscript.*

Point 1

there are so many people excluded at baseline, I think a characteristic comparison table is needed to compare those included and excluded

Response 1: *Thank you for your meaningful comment. We fully agree with your opinion that the large number of excluded participants could cause confusion. We have created a flow chart to illustrate the inclusion and exclusion of participants based on the criteria. Once again, thank you for your great effort in reviewing our manuscript and giving meaningful feedback.*

✓ ***Revised manuscript, Results:* Update Figure 1. Participation flow chart**

Fig 1. Flow chart of participant selection.

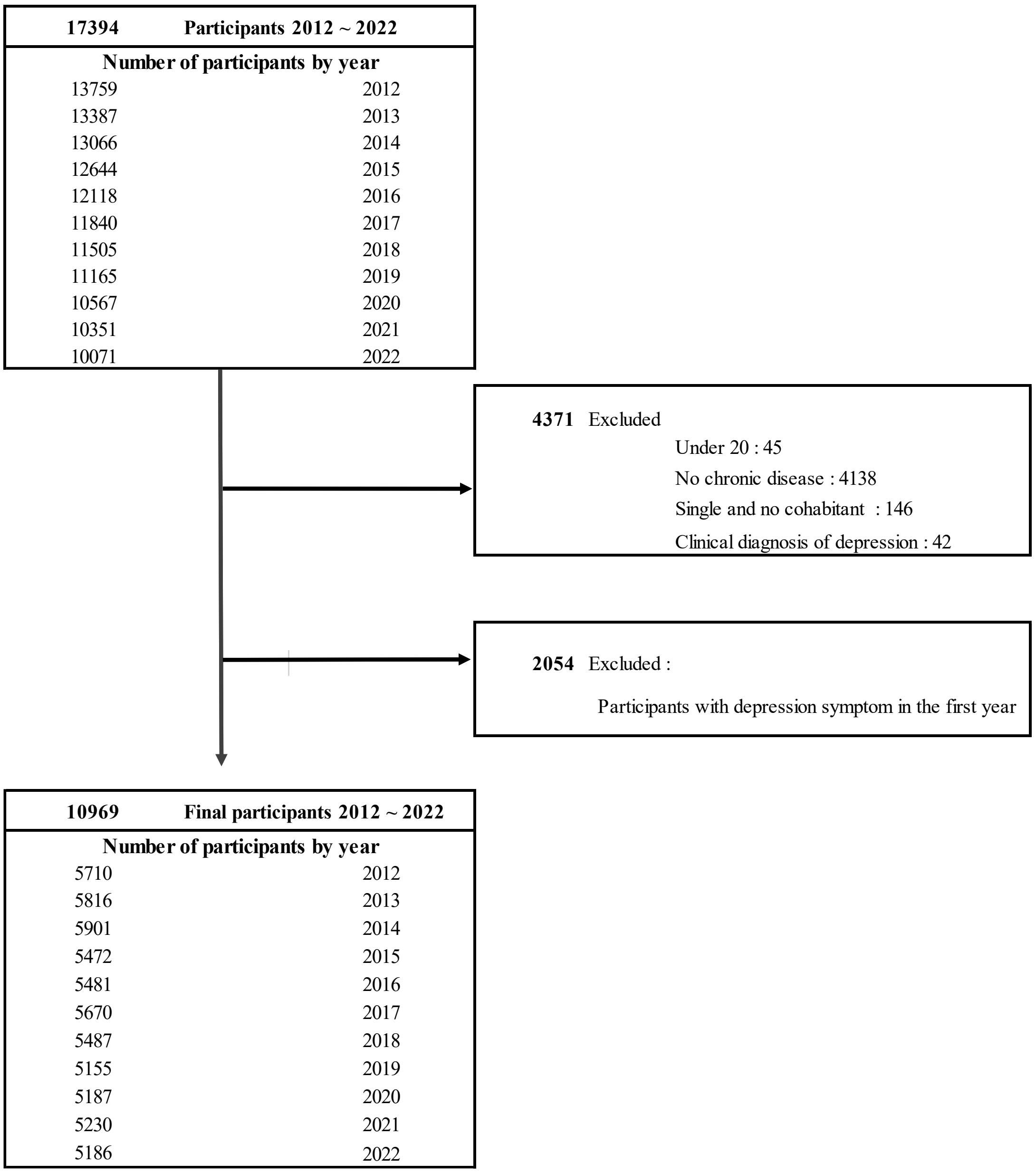

Point 2

why excluded those under 29?

Response 2: *Thank you for your comment. Our study focuses on individuals who are either married or living with family members and have chronic diseases. Considering the characteristics of chronic disease patients and previous studies, we initially selected participants aged 30 and above. However, taking into account the reviewer's comments and additional prior research, we have adjusted the participant age range to include those aged 20 and above. Teenagers were excluded as the incidence of chronic diseases is lower in this age group, and none of the teenagers in the panel had chronic diseases. Once again, thank you for your great effort in reviewing our manuscript and giving meaningful feedback.*

- ✓ ***Revised manuscript, line 93-99, page 4-5, Methods Data source and study population :*** ***For our study's data, the presence of chronic diseases was reported by participants themselves. We categorized participants as chronic disease patients if they reported having the condition for less than 3 months, between 3 months and less than 6 months, or more than 6 months [23]. We excluded participants from 2012 to 2022 who were younger than 20 years old, did not have chronic diseases, were not married or did not have cohabitants, were diagnosed with depression, or showed depressive symptoms in their first entry year in the panel. At baseline, there were a total of 5,816 participants. The average participation period for the panel participants was 5.4 years.***

Point 3

Is CESD-11 validated in Korean population with chronic diseases ?

Response 3: *Thank you for your positive feedback on our study. The CESD-11 is a shortened version of the CESD-20, which has been validated as a tool for screening depressive symptoms in a broad range of Korean populations, including those with disabilities. However, it has not been specifically validated for use solely among Korean individuals with chronic diseases. We will include this limitation in our study to acknowledge this aspect. We will include this limitation in the study.*

- ✓ ***<u>Revised manuscript, line 102-110, page 6, Methods Measures:</u> The dependent variable was depressive symptoms. The research employed the Center for Epidemiologic Studies Depression Scale (CESD-11) to assess depressive symptoms. The CESD-11, a concise version of the original 20-item scale, is a well-validated self-report screening instrument [24, 25]. The CESD-11 has been applied across a wide range of Korean populations, including those with disabilities [26, 27]. The total CESD-11 score is computed by summing the scores for all 11 questions and multiplying them by 20/11 [28, 29]. A score of 16 or higher indicates the presence of depressive symptoms. Scores falling within the range of 16–20 indicate mild depressive symptoms, scores between 20 and 24 indicate moderate depressive symptoms, and a score of 25 or higher indicates severe depressive symptoms.***

- ✓ ***<u>Revised manuscript, line 230-233, page 17, Discussion</u> : First, this study used the CESD-11 scale to assess depressive symptoms in individuals with chronic diseases. While the CESD-11 is widely used to identify depressive symptoms across a broad range of Korean populations, including individuals with disabilities, its validity specifically for individuals with chronic diseases has not been established***
- ✓ ***Added references***:

- Chon k-K, Rhee M-K. Preliminary Development of Korean Version of CES-D. Korean Journal of Clinical Psychology. 1992;11(1):65-76.

- Kang SH, Kim S, Park E-C, Jang S-I. Effects of material hardship on depression among adults in South Korea: insights from by the Korea Welfare Panel Study 2008–2017. International Journal for Equity in Health. 2021;20(1):202. doi: 10.1186/s12939-021-01531-1.

- Shin J, Choi JW, Jang S-i, Choi Y, Lee SG, Ihm TH, et al. The temporal association of excessive health expenditure with suicidal ideation among primary income earners: a cross-sectional design using the Korean Welfare Panel Survey (KoWePS). BMJ Open. 2015;5(6):e007421. doi: 10.1136/bmjopen-2014-007421.

- Kim JY. Does poverty matter for depression in people with disabilities? The

Mediating Effects of Life Satisfaction. Journal of Social Service Research. 2022;48(3):430-9.

- Ma S, Jeon B, Roh S-h, Kim C-S. Violence victimization experiences and depression among individuals with disabilities. Korean J Health Educ Promot. 2023;40(5):55-68.

Point 4

the scoring method for participants' responses to five statements are confusing: 1 point for "never " to 5 point for "always" (line 117), so the higher the total score, the more negative resolve method is, but why the opposite is the definition being stated?

Response 4: *Thank you for your meaningful feedback. We apologize for confusion caused by the different coding methods for each variable. We will explain further. The approach to handling family conflict is based on the scoring and evaluation method suggested by KOWEP. In this study, we evaluated how family members have interacted and resolved conflicts over the past year based on participants' subjective responses to five items. Participants responded to the items using a scale of "Not at all," "Somewhat disagree," "Neutral," "Somewhat agree," and "Strongly agree." Scores are assigned to each item based on the responses, and higher total scores indicate a more positive assessment. For the statements "There are frequent disagreements in my family," "My family members get so angry that they throw things," "My family members criticize each other," and "My family members hit each other," a response of "Not at all" is given 5 points, and a response of "Strongly agree" is given 1 point. For the statement "My family members discuss issues calmly," a response of "Not at all" is given 1 point, and a response of "Strongly agree" is given 5 points. The calculated values are then divided by 5 for evaluation. Through this comment, we could improve the completeness of the text. Thanks again your great effort in reviewing our manuscript.*

- ✓ ***Revised manuscript, line 111-122, page 6, Methods Measures:*** ***In this study, the main independent variable was the methods for managing family conflict, evaluated using the scoring and evaluation method recommended by KoWePS. We evaluated how family members have interacted and resolved conflicts over the past year based on***

participants' subjective responses to five items. Participants responded to the items using a scale of "Not at all," "Somewhat disagree," "Neutral," "Somewhat agree," and "Strongly agree." Scores are assigned to each item based on the responses, and higher total scores indicate a more positive assessment. For the statements "There are frequent disagreements in my family," "My family members get so angry that they throw things," "My family members criticize each other," and "My family members hit each other," a response of "Not at all" is given 5 points, and a response of "Strongly agree" is given 1 point. For the statement "My family members discuss issues calmly," a response of "Not at all" is given 1 point, and a response of "Strongly agree" is given 5 points. The calculated values are then divided by 5 for evaluation.

Point 5

What was the plot for on page 13? What is the interpretation of the results?

Response 5: *Thank you for your meaningful comment. We apologize for the confusion. The plot presented on page 13 examined the severity of each depression symptom in relation to family conflict resolution methods. However, we have decided to delete Table 5 based on feedback from other reviewers, as it was deemed unnecessary for our study. Thank you.*

Response to Reviewer #2's comments

In introduction point 1

How vulnerable people with chronic diseases are to depression, not depression.

In introduction response 1: *Thank you for your meaningful comment. We fully agree with your suggestions. Following your advice, we have removed the unnecessary paragraph about depressive symptoms in the first paragraph and instead added information about the vulnerability of individuals with chronic diseases to depressive symptoms. Once again, thank you for your great effort in reviewing our manuscript and providing meaningful feedback.*

- ✓ ***Revised manuscript, line 53-63, page 3, Introduction:* People with chronic conditions have limited activity or persistent pain, which reduces opportunities for activity[1]. These characteristics of chronic disease increase the risk of physical[2], mental[3, 4], and psychological distress and lower life satisfaction in people with chronic disease when compared to physically healthy people[5]. Additionally, although stress varies by individual characteristics, patients with higher stress levels due to the general characteristics of chronic disease are more likely to experience mental disorders[6]. Similarly, people with multiple chronic conditions are at a higher risk for depressive symptoms, which is the focus of our study, than people with a single chronic condition [7-9]. Managing depression and its symptom is important because depression can lead to additional risks of social isolation[10], additional psychiatric disease, and suicidal ideation[11]. Therefore, managing the condition of chronic disease patients who are more vulnerable to depressive symptoms is extremely important.**
- ✓ ***Added references*:**
    - Verhaak PFM, Heijmans MJWM, Peters L, Rijken M. Chronic disease and mental disorder. Social Science & Medicine. 2005;60(4):789-97. doi: https://doi.org/10.1016/j.socscimed.2004.06.012.
    - Tsai M-H, Wu Y-H, Bevel MS. The relationship of chronic disease conditions to mental and physical health among cancer survivors. Supportive Care in Cancer. 2023;31(6):364.
    - Moussavi S, Chatterji S, Verdes E, Tandon A, Patel V, Ustun B. Depression,

chronic diseases, and decrements in health: results from the World Health Surveys. The Lancet. 2007;370(9590):851-8. doi: https://doi.org/10.1016/S0140-6736(07)61415-9.

- Chapman DP, Perry GS, Strine TW. The vital link between chronic disease and depressive disorders. Prev Chronic Dis. 2005;2(1):A14. Epub 2005/01/27. PubMed PMID: 15670467; PubMed Central PMCID: PMCPMC1323317.

- Van Wilder L, Vandepitte S, Clays E, Devleesschauwer B, Pype P, Boeckxstaens P, et al. Psychosocial factors associated with health-related quality of life in patients with chronic disease: Results of a cross-sectional survey. Chronic Illness. 2023;19(4):743-57. doi: 10.1177/17423953221124313. PubMed PMID: 36069001.

- Heijmans M, Rijken M, Foets M, de Ridder D, Schreurs K, Bensing J. The stress of being chronically ill: from disease-specific to task-specific aspects. Journal of behavioral medicine. 2004;27:255-71.

- Harpole LH, Williams JW, Olsen MK, Stechuchak KM, Oddone E, Callahan CM, et al. Improving depression outcomes in older adults with comorbid medical illness. General Hospital Psychiatry. 2005;27(1):4-12. doi: https://doi.org/10.1016/j.genhosppsych.2004.09.004.

- Alan K. Koike, M.D., M.S.H.S. ,, Jürgen Unützer, M.D., M.P.H. , and, Kenneth B. Wells, M.D., M.P.H. Improving the Care for Depression in Patients With Comorbid Medical Illness. American Journal of Psychiatry. 2002;159(10):1738-45. doi: 10.1176/appi.ajp.159.10.1738. PubMed PMID: 12359681.

- Ma Y, Xiang Q, Yan C, Liao H, Wang J. Relationship between chronic diseases and depression: the mediating effect of pain. BMC Psychiatry. 2021;21(1):436. doi: 10.1186/s12888-021-03428-3.

- Christiansen J, Lund R, Qualter P, Andersen CM, Pedersen SS, Lasgaard M. Loneliness, Social Isolation, and Chronic Disease Outcomes. Annals of Behavioral Medicine. 2020;55(3):203-15. doi: 10.1093/abm/kaaa044.

- Cogley C, Bramham J, Bramham K, Smith A, Holian J, O'Riordan A, et al. High rates of psychological distress, mental health diagnoses and suicide attempts in people with chronic kidney disease in Ireland. Nephrology Dialysis Transplantation. 2023;38(10):2152-9. doi: 10.1093/ndt/gfad021.

In methods point 1

Isn't the participants for people under 30 years old? Line 101 needs to be checked.

In methods response 1: *Thank you for your comment. We apologize for the confusion. Considering the characteristics of chronic disease patients and previous studies, we initially selected participants aged 30 and above. However, taking into account the reviewer's comments and additional prior research, we have adjusted the participant age range to include those aged 20 and above. Teenagers were excluded as the incidence of chronic diseases is lower in this age group, and none of the teenagers in the panel had chronic diseases. Once again, thank you for your great effort in reviewing our manuscript and giving meaningful feedback.*

- ✓ ***Revised manuscript, line 93-99, page 4-5, Methods Data source and study population :* For our study's data, the presence of chronic diseases was reported by participants themselves. We categorized participants as chronic disease patients if they reported having the condition for less than 3 months, between 3 months and less than 6 months, or more than 6 months [23]. We excluded participants from 2012 to 2022 who were younger than 20 years old, did not have chronic diseases, were not married or did not have cohabitants, were diagnosed with depression, or showed depressive symptoms in their first entry year in the panel. At baseline, there were a total of 5,816 participants. The average participation period for the panel participants was 5.4 years.**

In methods point 2

The author should further explain what chronic diseases are.

In methods response 2: *Thank you for your comment. Thank you for your comment. We fully agree with the suggestion to provide more explanation regarding chronic diseases. In the case of chronic diseases, the data were collected from the Korean Welfare Panel Study, where major diseases were coded to classify chronic diseases and verified through self-reports by participants. There is a total list of 32 chronic diseases. Even if participants had multiple chronic conditions, they were required to select the one primary chronic disease they considered most significant. Accordingly, we have included the top 5 most frequently reported chronic diseases in the table. Participants with depression were excluded from this study;*

therefore, the remaining 26 chronic diseases are categorized as 'Others'. Additional information regarding these 26 chronic diseases has been provided in Supplementary 1.

✓ ***Revised manuscript, Results:* Update Supplementary 1**

Supplementary1. General characteristics of the baseline population with chronic diesase

Variables		Depressive symptoms (CESD-11 ≥ 16)						
		Total		No		Yes		*P*-value
		N	%	N	%	N	%	
		**5,816**	**100.0**	**5,112**	**87.9**	**704**	**12.1**	
**Chronic disease**								<.0001
	Hypertension	1,604	27.6	1,443	90.0	161	10.0	
	Arthritis,lumbago, sciatica, disc disease	1,444	24.8	1,218	84.3	226	15.7	
	Other diseases (acute diseases, etc.)	305	5.2	281	92.1	24	7.9	
	Diabetes	632	10.9	549	86.9	83	13.1	
	Cancer (stomach, liver, lung, bronchi, etc.)	205	3.5	171	83.4	34	16.6	
	Gastritis, peptic ulcer, duodenal ulcer, etc.	204	3.5	184	90.2	20	9.8	
	Chronic hepatitis, liver cirrhosis	61	1.0	58	95.1	3	4.9	
	Dental caries	213	3.7	210	98.6	3	1.4	
	Thyroid disease	111	1.9	104	93.7	7	6.3	
	Stroke, cerebrovascular disease	128	2.2	95	74.2	33	25.8	
	Myocardial infarction, angina pectoris	203	3.5	165	81.3	38	18.7	
	Pulmonary tuberculosis, tuberculosis	17	0.3	16	94.1	1	5.9	
	Chronic bronchitis (severe sputum, cough)	19	0.3	15	78.9	4	21.1	
	Asthma	68	1.2	61	89.7	7	10.3	
	Cataract, glaucoma	43	0.7	39	90.7	4	9.3	
	Chronic otitis media	9	0.2	9	100.0	0	0.0	
	Chronic renal failure (chronic kidney disease)	43	0.7	33	76.7	10	23.3	
	Fracture, osteoporosis and sequelae due to accidents	70	1.2	63	90.0	7	10.0	
	Osteoporosis	87	1.5	75	86.2	12	13.8	
	Anemia	18	0.3	15	83.3	3	16.7	
	Hyperlipidemia	56	1.0	50	89.3	6	10.7	
	Hernia (inguinal hernia)	19	0.3	19	100.0	0	0.0	
	Chronic sinusitis (sinusitis)	7	0.1	7	100.0	0	0.0	
	Bronchiectasis	5	0.1	5	100.0	0	0.0	
	Allergic rhinitis	88	1.5	86	97.7	2	2.3	
	Temporomandibular joint disorder	2	0.0	2	100.0	0	0.0	
	Atopic dermatitis	44	0.8	41	93.2	3	6.8	
	Urinary incontinence	1	0.0	0	0.0	1	100.0	
	Chronic periodontal disease (gingivitis, gum disease)	68	1.2	64	94.1	4	5.9	
	Rare and intractable diseases	36	0.6	28	77.8	8	22.2	
	Hypotension	6	0.1	6	100.0	0	0.0	

In methods point 3

Didn't investigate the duration of the prevalence of chronic diseases?

In methods response 3: *Thank you for your comment. The presence of chronic diseases was reported by participants themselves. Referring to previous studies targeting individuals with chronic conditions, we categorized participants as chronic disease patients if they reported having the condition for less than 3 months, between 3 months and less than 6 months, or more than 6 months. Once again, thank you for your great effort in reviewing our manuscript and giving meaningful feedback.*

- ✓ ***Revised manuscript, line 96-96, page 5, Methods Data source and study population:*** ***For our study's data, the presence of chronic diseases was reported by participants themselves. We categorized participants as chronic disease patients if they reported having the condition for less than 3 months, between 3 months and less than 6 months, or more than 6 months [23].***
- ✓ ***Added references:***
  - *Han K-M, Ko Y-H, Yoon H-K, Han C, Ham B-J, Kim Y-K. Relationship of depression, chronic disease, self-rated health, and gender with health care utilization among community-living elderly. Journal of Affective Disorders.*

In results point 1

table 5, It would be better to present either the table or the figure, not both.

In results response 1:

Thank you for your comment. We share the same opinion. Another reviewer also mentioned that Table 5 diverged from the main argument. Therefore, we will delete Table 5. Thank you.

In discussion point 1

I don't understand the sentence. Please organize your thoughts and write them down again. The arguments before and after do not match.

In discussion response 1: *Thank you for your comment. We have revised and reorganized the entire discussion section. We also made changes to parts that could potentially cause confusion to the argument due to inconsistencies in the beginning and end sections.*

- ✓ ***Revised manuscript, line 199-215, page 16, Discussion:*** ***Our study found an association between depressive symptoms and changes in how patients with chronic diseases resolve family conflicts. A change in the negative approach to resolving conflict and no change in the negative approach were linked to higher odds of experiencing depressive symptoms compared with the positive approach. By contrast, participants who changed from a negative to a positive method showed lower levels of depressive symptoms than those who continued using a negative method of conflict resolution. Consistent with previous studies, our results indicated that conflict within family relationships influenced depressive symptoms, and addressing conflicts is crucial for managing such symptoms [30, 31].***

  Our study revealed that economically active participants with chronic diseases faced higher odds of depressive symptoms when their family conflict resolution methods remained persistently negative. This finding aligns with existing research indicating that individuals with chronic diseases are more susceptible to presenteeism [32] and psychological stress in the workplace compared to healthy individuals [33], which can spill over into the home, exacerbating family relationship conflicts [34].

Therefore, addressing mental health issues for individuals with chronic diseases is crucial in both workplace and home settings [35]. Our study findings are thus corroborated by those of other studies. This underscores the need for continuous attention and support to improve the overall mental health of individuals with chronic diseases.

✓ ***Added references:***

- Holland P, Collins AM. "Whenever I can I push myself to go to work": a qualitative study of experiences of sickness presenteeism among workers with rheumatoid arthritis. Disability and Rehabilitation. 2018;40(4):404-13. doi: 10.1080/09638288.2016.1258436.

- McGonagle AK, Barnes-Farrell JL. Chronic Illness in the Workplace: Stigma, Identity Threat and Strain. Stress and Health. 2014;30(4):310-21. doi: https://doi.org/10.1002/smi.2518.

- Frone MR, Russell M, Cooper ML. Relation of work–family conflict to health outcomes: A four-year longitudinal study of employed parents. Journal of Occupational and Organizational Psychology. 1997;70(4):325-35. doi: https://doi.org/10.1111/j.2044-8325.1997.tb00652.x.

- Sorensen G, Landsbergis P, Hammer L, III BCA, Linnan L, Yancey A, et al. Preventing Chronic Disease in the Workplace: A Workshop Report and Recommendations. American Journal of Public Health. 2011;101(S1):S196-S207. doi: 10.2105/ajph.2010.300075. PubMed PMID: 21778485.

In discussion point 2

Please write down the line number.

In discussion response 2: *Thank you for your comment. We apologize for the confusion. We have regenerated line numbers in the text. Thank you.*

In conclusion point 1

Please check the sentence and write it down concisely.

In conclusion response 1: *Thank you for your comment. We agree that the sentences in the conclusion need to be simplified. Since some sentences repeat the same points, we deleted unnecessary sentences and emphasized the need for programs or services that offer positive problem-solving methods for chronic patients and their families through our research findings. Thank you for the good suggestion.*

- ✓ ***Revised manuscript, line 248-257, page 18, Conclusion:*** ***Our study examined the relationship between family conflict resolution methods and depression in individuals over the age of 30 in Korea living with chronic diseases. The study found that using negative methods to resolve family conflicts was associated with symptoms of depression. There was a heightened risk of depressive symptoms when negative communication or physical violence was involved. Based on these results, programs and services must be developed to promote healthy conflict resolution for individuals and families, minimize negative change, and facilitate positive change. To enhance the study's scope, future research should incorporate different variables and effective conflict resolution strategies. Furthermore, it is important to compare and validate these findings with those of similar studies conducted in diverse cultural contexts to enhance the applicability of the research outcomes.***

In conclusion point 2

Please check the completion of the sentence in the first paragraph.

In conclusion response 2 : *Thank you for your comment. We apologize for the confusion. We have reorganized what we wanted to convey in the first sentence. We were able to improve our manuscript based on the meaningful comments that you have given us. Once again, we would like to offer our sincerest apologies for this confusion.*

- ✓ ***Revised manuscript, line 248-249, page 18, Conclusion:*** ***Our study examined the***

relationship between family conflict resolution methods and depression in individuals over the age of 30 in Korea living with chronic disease.
